# Dissecting how modular polyketide synthase ketoreductases interact with acyl carrier protein-attached substrates†
†Electronic supplementary information (ESI) available: Complete experimental details and additional figures. See DOI: 10.1039/c7cc04625a


**DOI:** 10.1039/c7cc04625a

**Published:** 2017-10-05

**Authors:** Luisa Moretto, Steven Vance, Brennan Heames, R. William Broadhurst

**Affiliations:** a Department of Molecular Biosciences , The University of Texas at Austin , Austin , TX 78712 , USA; b Crescendo Biologics Ltd , Meditrina Building 260 , Babraham Research Campus , Cambridge CB22 3AT , UK; c Department of Biochemistry , University of Cambridge , 80 Tennis Court Road , Cambridge CB2 1GA , UK . Email: rwb1002@cam.ac.uk

## Abstract

Interaction studies using fragments excised from the modular mycolactone polyketide synthase show that ketoreductase domains possess a generic binding site for acyl carrier protein domains and provide evidence that the pendant 5′-phosphopantetheine prosthetic group plays a key role in delivering acyl substrates to the active site in the correct orientation.

## Abstract 2

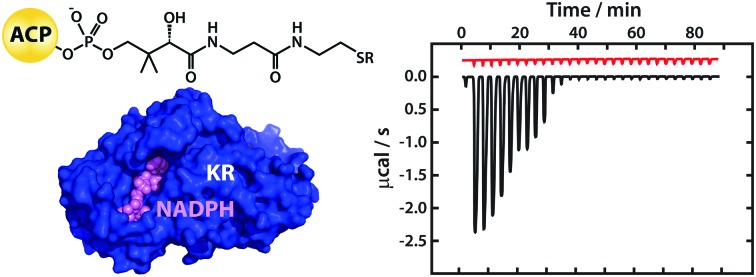
Interaction studies show that KR domains possess a generic binding site for ACP domains and provide evidence that the 5′-phosphopantetheine prosthetic group plays a key role in delivering acyl substrates to the active site in the correct orientation.

## 

The carbon scaffolds of many clinically important natural products are constructed in an assembly-line fashion by modular type I polyketide synthases (PKSs), protein complexes that comprise multiple modules of covalently-linked catalytic domains.^1^ Polyketide chains are assembled from coenzyme A-attached α-carboxyacyl extender units selected by the acyltransferase (AT) domain in each module, loaded onto the 4′-phosphopantetheine (Ppant) arm of an acyl carrier protein (ACP) domain and then linked with the growing polyketide chain in a decarboxylative condensation reaction catalysed by a ketosynthase (KS) domain. Each module performs a separate chain extension cycle, during which accessory domains with ketoreductase (KR), dehydratase (DH) and enoyl reductase (ER) activities may, if present, adjust the oxidation state of the β-keto group. The substrate is then passed onwards to the KS domain of the next module or, in the case of a final module, is released from the ACP, for example by a C-terminal thioesterase (TE) domain.

The stereochemical complexity of bioactive polyketides is crucial for targeting specific receptors. In particular, hydroxyl substituents can take part in directional hydrogen bonding interactions or act as points of attachment for additional functional groups.^2^ Hydroxyl groups are introduced into polyketide chains by KR domains, which comprise Rossman fold structural and catalytic subdomains^3^ that use NADPH to reduce substrate β-keto sites to secondary alcohols.^4^ A conserved triad of tyrosine, serine and lysine side-chains in the active sites of A- and B-type KR domains creates β-hydroxyl groups with either 3*S* or 3*R* orientations, respectively (Scheme 1).^5^ In addition, class 2 (*i.e.* A2- and B2-type) domains deploy the same set of catalytic residues to generate 2*S* stereochemistry at adjacent alkyl-substituted α-sites.^6^ By contrast, A1- and B1-type KR domains leave α-substituents unchanged in the 2*R* orientation.^7^

**Scheme 1:**
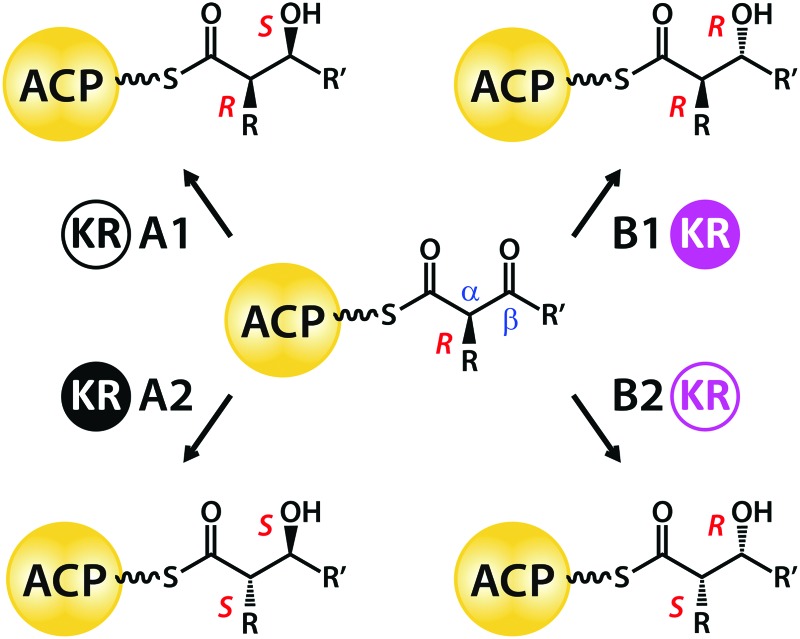
Stereocontrol exerted by type I modular PKS KR domain variants on ketoreduction at β-sites and on epimerization at α-sites.

A1-, A2-, B1- and B2-type KR domains bind their NADPH cofactor in a common orientation^3^ and all these variants use only the 4-*pro-S* hydride of nicotinamide for ketoreduction of substrates.^8^ Thus, selective attack at the *Re* or *Si* face of a β-ketoacyl intermediate to generate a hydroxyl group with 3*S* or 3*R* stereochemistry, respectively, must depend on the substrate being presented to the active site in different orientations.^5^ B-type KR domains appear to use the leucine side-chain of a conserved Leu-Asp-Asp signature to guide Ppant-attached β-ketoacyl substrates into the active site groove.^7^,^9^,^10^ A-type domains lack this steering motif, but instead possess a conserved tryptophan side-chain on the other side of the cleft, whose role is not yet understood; the binding of cofactor promotes closure of a “lid-loop” feature that restricts active site access by the substrate to the opposite direction.^11^

Most investigations into the stereochemistry of ketoreduction have been conducted using synthetic β-ketoacyl thioester substrates linked to *N*-acetyl cysteamine (**7**; Scheme 2), intended to mimic the final segment of the Ppant prosthetic group joined to the *holo* form of an ACP domain (**1a**).^12^ This approach was validated by the finding of near identical kinetic parameters when the KR domain from module 1 of the 6-deoxyerythronolide B synthase (DEBS KR1) was challenged with a diketide substrate attached either to **7** or to *R*-pantetheine (**2**),^13^ independent of whether the KR was excised from or functioned within an intact module.^14^ Further, experiments with standalone constructs demonstrate that KR domains are equally successful at reducing β-ketoacyl substrates when delivered by an ACP fragment from the same (cognate) module, from a non-cognate module of the same PKS system, or even from a different system.^15^ Nevertheless, both the mode of substrate presentation and the modular context of the KR domain have been found to exert subtle effects on product stereoselectivity and specificity.^16^–^18^ In addition, despite systematic experimentation, the configurations of product α-alkyl and β-hydroxyl groups cannot yet be manipulated reliably *in vivo* simply by swapping the sequence of a heterologous KR domain with desirable reaction preferences into a given PKS module.^2^,^19^ One rationale for this unpredictability is that interactions between a ketoreductase and other domains within the same module may perturb the outcome.^17^,^20^ Despite this wealth of indirect evidence for the finely balanced nature of inter-domain interactions, we have lacked direct quantitative data on the strength of binding between KR and ACP, and about the contribution to this of the Ppant prosthetic group.

**Scheme 2:**
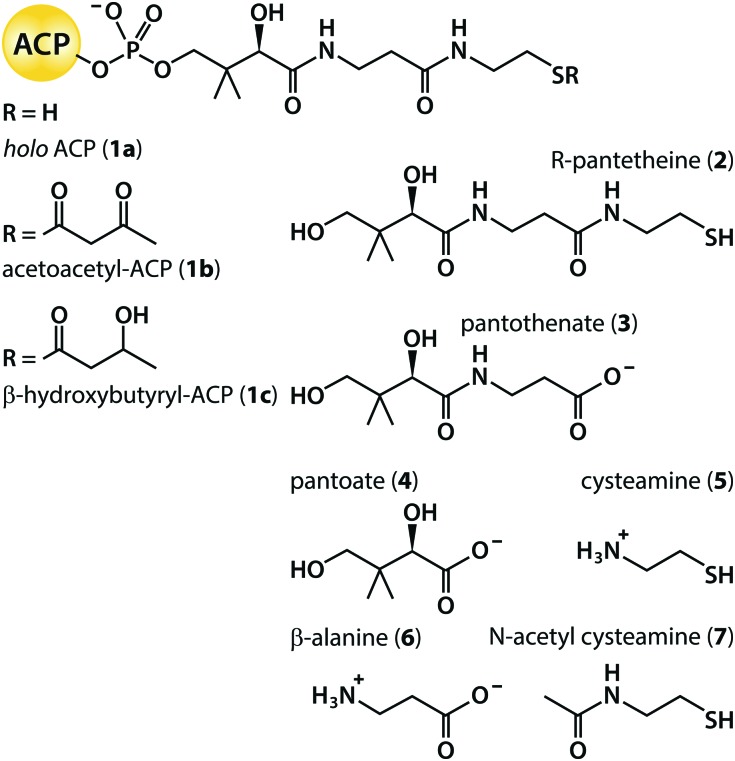
ACP species and Ppant fragment mimics.

To investigate the interactions of KR and ACP domains, we selected as a model system the giant PKS responsible for producing the toxin mycolactone in *Mycobacterium ulcerans*. This PKS comprises three polypeptide chains: MLSA1, MLSA2 and MLSB, encoded on the plasmid pMUM001 (ESI,† Fig. S1).^21^ The 16 extension modules make use of only two varieties of KR domain, A1 and B1. Unusually, the amino acid sequences of all KR domains of the same type are identical, even though their substrates vary significantly; by contrast, the A1 and B1 species share a more typical sequence identity of 38%.^22^ We cloned, expressed and purified recombinant protein fragments spanning selected A1- and B1-type KR domain sequences (mKRa from module 5 of MLSA1 and mKRb from module 7 of MLSB) and also their cognate ACP domains (mACPa and mACPb).

On injecting the *apo* form of mACPa (which lacks the Ppant prosthetic group) into a solution of mKRa in the presence of excess NADPH, isothermal titration calorimetry (ITC)^23^ detected a series of exothermic heat changes, confirming the formation of a protein complex (Fig. 1A). Integration of the thermogram yielded a sigmoidal isotherm plot consistent with 1 : 1 binding stoichiometry and a dissociation constant (*K*_D_) of 2.5 μM (Table 1). Titration of *apo* mACPb into a solution of mKRb in the presence of NADPH (Fig. 1B) gave comparable results (*K*_D_ 2.6 μM), suggesting that these KR domains may interact with their cognate ACPs in similar ways. This hypothesis was tested by titrating the non-cognate species *apo* mACPb and *apo* mACPa against mKRa and mKRb, respectively, in excess NADPH; the resulting *K*_D_ values barely differed from those observed for cognate interactions (Table 1). In the same way, replacing NADPH with its oxidised form NADP^+^ only marginally altered the affinity of *apo* mACPb for mKRb (Table 1). These results show that KR/cofactor complexes possess an interface region that can interact with an ACP domain. The redox state of the cofactor has little effect on affinity for the ACP, suggesting that this change does not perturb the binding surface. The interface appears not to discriminate between cognate and non-cognate *apo* ACP species and therefore must recognize conserved features that are common to both types of domain. Micromolar *K*_D_ values imply that the interaction between these ACP and KR fragments is relatively weak. However, within a module, covalent tethering of the domains by a flexible polypeptide linker will boost the effective concentration of one partner with respect to the other into the millimolar range and thus promote binding.^24^

**Fig. 1:**
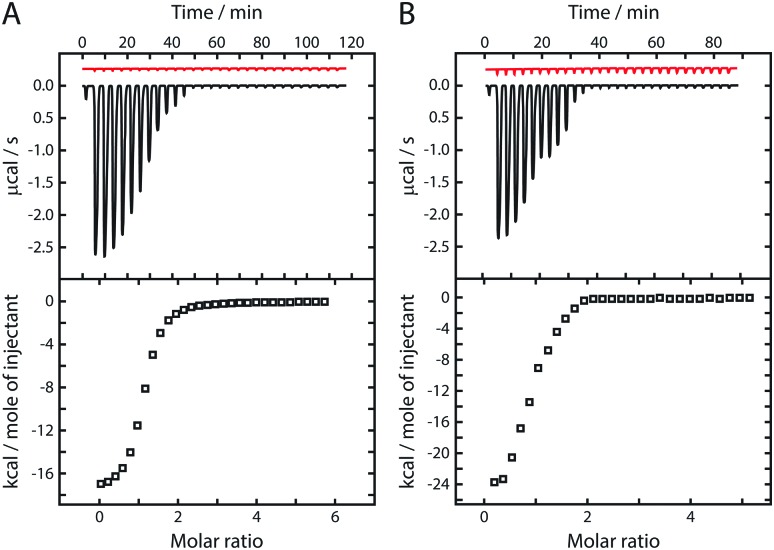
Representative ITC thermograms (upper panels) and isotherm plots (lower panels), showing consecutive injections of (A) *apo* mACPa against mKRa and (B) *apo* mACPb against mKRb, in the presence of excess NADPH. Thermogram traces for dilution control experiments are displayed in the upper panels in red.

**Table 1:** Dissociation constants for interactions between ketoreductase domains and chemically modified ACP species

Ligand	Cofactor	*K*_D_ with mKRa^*a*^/μM	*K*_D_ with mKRb^*a*^/μM
*apo* mACPa	NADPH	2.50 ± 0.20	2.20 ± 0.55
*apo* mACPb	NADPH	1.80 ± 0.26	2.60 ± 0.40
*apo* mACPb	NADP^+^	—	2.20 ± 0.62
*holo* mACPa (**1a**)	NADPH	0.77 ± 0.07	0.63 ± 0.05
*holo* mACPb (**1a**)	NADPH	0.74 ± 0.06	0.61 ± 0.23
*holo* mACPb (**1a**)	NADP^+^	—	0.75 ± 0.11
Acetoacetyl-mACPa (**1b**)	NADP^+^	0.43 ± 0.03	—
Acetoacetyl-mACPb (**1b**)	NADP^+^	—	0.38 ± 0.02
β-Hydroxybutyryl-mACPa (**1c**)	NADPH	0.29 ± 0.07	—
β-Hydroxybutyryl-mACPb (**1c**)	NADPH	—	0.27 ± 0.01

^*a*^Values represent the average and standard deviation over three repeat ITC experiments.

Next, to assess the consequences of covalent attachment of the Ppant prosthetic group, we titrated the *holo* (**1a**) forms of mACPa and mACPb against mKRa and mKRb, respectively, in excess NADPH (Table 1). Exothermic heat changes consistent with 1 : 1 binding stoichiometry were detected in both cases, but the resulting dissociation constants (*K*_D_ 0.6 to 0.8 μM) were noticeably smaller than those obtained for *apo* ACP species. Again, substituting non-cognate for cognate *holo* ACP species, or NADP^+^ for NADPH, had only minor effects on the affinity of the KR/cofactor/ACP interaction (Table 1). We take this modest but consistent reduction in *K*_D_ values as evidence that the Ppant arm plays a meaningful role in extending the interface between the ACP domain and the KR/cofactor binary complex.

To investigate the consequences of acyl group tethering, we prepared acetoacetyl (**1b**) and β-hydroxybutyryl (**1c**) derivatives of mACPa and mACPb. Acetoacetyl-ACP species were selected to mimic the substrate for reduction, so these were studied in the presence of excess NADP^+^ to prevent the forward reaction from occurring. Similarly, excess NADPH was used in studies of β-hydroxybutyryl-ACP product mimics, to prevent the reverse reaction. ITC experiments involving substrate or product mimics and their cognate KR domains yielded dissociation constants in the range 0.3 to 0.4 μM (Table 1). Acyl-ACP substrates therefore bind their KR domains with two-fold higher affinity than *holo* ACP forms, which themselves interact with three-fold greater affinity than the corresponding *apo* species. The tighter binding of acyl-ACP species indicates that the substrate and product mimics make additional favourable contributions to the free energy of formation of the KR/cofactor/ACP tertiary complex, consistent with ketoreductase active sites having evolved to accommodate similar structures. Since KR domains are typically capable of catalysing either reduction or oxidation reactions depending on the [NADPH] : [NADP^+^] ratio, it is not surprising that the affinities for substrate and product mimics should be so similar.

The experiments described above demonstrate that the interaction between an acyl-ACP species and a KR/cofactor binary complex is multivalent, comprising distinct contributions from the ACP surface, the prosthetic group and the thioester-linked substrate. However, the increase in affinity observed when the Ppant and acyl moieties are included is relatively small, indicating that the entropic penalties engendered by restricting the motion of the freely swinging prosthetic group arm^25^ are only just compensated for by the favourable enthalpy change resulting from extension of the binding interface. The net effect is likely to be an increase in the specificity of the interaction (due to contacts with multiple binding sites), but with only minor effects on the dissociation constant.^26^ This moderation of high affinity binding could facilitate enzyme turnover by minimizing the opportunity for substrate or product inhibition.

Finally, to distinguish between contributions made by different components of the prosthetic group, we screened the binding properties of a panel of commercially available small molecule fragments of 4′-phosphopantetheine (Scheme 2). Titration of *R*-pantetheine (**2**) against either mKRa or mKRb in the presence of excess NADPH or NADP^+^ yielded exothermic heat change profiles with 1 : 1 binding stoichiometry and dissociation constants close to 1.2 μM (Table 2). Consistent *K*_D_ values across all four KR/cofactor combinations were also returned for pantothenate (**3**; 0.7 μM) and for *N*-acetyl cysteamine (**7**; 1.1 μM). All three fragments bound to mKRa and mKRb with affinities similar to those reported above for *holo* ACP species (**1a**). Covalent attachment of an ACP domain to a pantetheine moiety *via* a phosphate group therefore causes only a minor increase in affinity for the KR/cofactor binary complex compared to interactions with the separate components (*K*_D_ 1.8 to 2.6 μM for *apo* ACPs; *K*_D_ 1.2 μM for **2**). If it can be assumed that Ppant fragments dock into the same sites that they bind when present in the *holo* form, this suggests that interactions with the ACP surface and with the prosthetic group are of similar importance. Further, the *K*_D_ values for **2** and its pantothenate and *N*-acetyl cysteamine sub-sections are not much different, implying once again a delicate balance between entropic and enthalpic contributions to the free energy change on binding.

**Table 2:** Dissociation constants for interactions between ketoreductase domains and Ppant fragment mimics

Ligand	Cofactor	*K*_D_ with mKRa^*a*^/μM	*K*_D_ with mKRb^*a*^/μM
*R*-Pantetheine (**2**)	NADPH	1.10 ± 0.04	1.20 ± 0.30
*R*-Pantetheine (**2**)	NADP^+^	1.23 ± 0.16	1.20 ± 0.20
Pantothenate (**3**)	NADPH	0.70 ± 0.13	0.70 ± 0.10
Pantothenate (**3**)	NADP^+^	0.77 ± 0.06	0.60 ± 0.07
Pantoate (**4**)	NADPH	>1000	0.60 ± 0.10
Pantoate (**4**)	NADP^+^	>1000	0.45 ± 0.10
Cysteamine (**5**)	NADPH	>1000	>1000
Cysteamine (**5**)	NADP^+^	>1000	>1000
β-Alanine (**6**)	NADPH	0.97 ± 0.04	0.50 ± 0.09
β-Alanine (**6**)	NADP^+^	0.95 ± 0.05	0.50 ± 0.06
*N*-Acetyl cysteamine (**7**)	NADPH	1.03 ± 0.02	1.40 ± 0.30
*N*-Acetyl cysteamine (**7**)	NADP^+^	1.15 ± 0.12	0.94 ± 0.20

^*a*^Values represent the average and standard deviation over three repeat ITC experiments.

Titrations with **7** gave rise to initial heat changes in the range –1.0 to –2.0 μcal s^–1^ per injection, but the responses observed for cysteamine (**5**) were much weaker (–0.1 μcal s^–1^ per injection), resulting in isotherm plots that did not permit accurate curve fitting (see ESI†); in Table 2, results of this sort are attributed *K*_D_ values >1000 μM. Taking into account the small change in molecular structure between **5** and **7**, we interpret their ITC signatures as evidence that the truncated cysteamine fragment has lost the ability displayed by **7** of binding to KR/cofactor complexes. This difference could result from a change in species charge: at pH 7.5, the primary amine of **5** will be protonated, whereas the amide moiety of **7** will be uncharged.

ITC experiments with the two remaining panel members revealed distinct preferences regarding their interactions with mKRa and mKRb. Pantoate (**4**) and β-alanine (**6**) bound mKRb with similar affinity (*K*_D_ ∼ 0.6 μM); by contrast, mKRa bound **6** with slightly lower affinity (*K*_D_ 1.0 μM) and **4** very weakly indeed (*K*_D_ > 1000 μM). Finding that the β-alanine and pantoate fragments interact differently with A1- and B1-type KR domains is consistent with the full-length Ppant arm being able to adopt distinct binding modes in each case, so as to present an acyl substrate to the active site in the correct configuration.^5^,^7^

In summary, these results provide direct quantitative insight into the energetics of the KR/cofactor/ACP interaction in a typical extension module of a modular type I polyketide synthase. Our findings help to rationalize the previously observed stereochemical preferences of α-methyl β-ketothioester substrates connected to *N*-acetyl cysteamine (**7**), thioethyl-acetate or ethanethiol handles.^18^,^27^ They are also consistent with evidence from previous studies of KR/cofactor/ACP interactions in type II fatty acid synthase and PKS systems (where each enzyme activity is located on a separate polypeptide chain). Using surface plasmon resonance and tryptophan fluorescence methods, *K*_D_ values of 1.6 μM and 0.2 μM were measured for *apo*^28^ and *holo* ACP species,^29^ respectively, in good agreement with the results presented in Table 1. In all of these systems, optimal operation of the assembly line should be promoted by using additional favourable interactions with the prosthetic group to increase selectivity rather than to enhance the binding affinity, which would lengthen the residence time of the ACP domain.

The authors would like to thank the Wellcome Trust (grant number 094252/Z/10/Z) for funding this research. LM was supported by an EPSRC PhD studentship. We are grateful to Dr Katherine Stott for assistance with the ITC experiments, Dr Len Packman for help with mass spectrometry and Professor Peter Leadlay for a critical reading of the manuscript.

## Supplementary Material

Supplementary information
