# Supplementary material for: Dissecting how modular polyketide synthase ketoreductases interact with acyl carrier protein-attached substrates
Source: Chem Commun (Camb). 2017 Oct 5;53(83):11457–60. doi: 10.1039/c7cc04625a (PMC6038798; doi:10.1039/c7cc04625a)
Supplement: Supplementary file 1 [file CC-053-C7CC04625A-s001.pdf]

Electronic Supplementary Material (ESI) for ChemComm.
This journal is © The Royal Society of Chemistry 2017

Supplementary Information

Dissecting how modular polyketide synthase ketoreductases interact with acyl carrier protein-attached substrates

Luisa Moretto, Steven Vance, Brennan Heames and R. William Broadhurst

1. Materials, DNA isolation and manipulation 2
2. Protein expression and purification of KR domain samples 2
3. Protein expression and purification of *apo* ACP samples 3
4. Validation of KR domain secondary structure and reductase activity 4
5. Preparation of *holo* and acyl-loaded ACP samples 5
6. Isothermal titration calorimetry experiments 6
7. References 19

Table S1: Oligonucleotide primers 8
Table S2: Amino acid sequences of protein constructs 8
Table S3: ESI-MS analysis of ACP species 9
Table S4: Kinetic parameters for KR domain constructs 9

Figure S1: Module organization for the mycolactone PKS system 10
Figure S2: SDS-PAGE analysis 11
Figure S2: ESI-MS spectra of *apo* and *holo* ACP species 12
Figure S4: ESI-MS spectra of acyl-ACP species 13
Figure S5: Circular dichroism spectra of KR domain constructs 14
Figure S6: Isothermal titration calorimetry data I 15
Figure S7: Isothermal titration calorimetry data II 16
Figure S8: Isothermal titration calorimetry data II 17
Figure S9: Isothermal titration calorimetry data IV 18

1. Materials, DNA isolation and manipulation

Restriction endonucleases and Taq ligase were obtained from New England BioLabs. Phusion DNA polymerase was purchased from Thermo Scientific. T5 exonuclease was purchased from Epicentre. T4DNA ligase, alkaline phosphatase and protease inhibitors were purchased from Roche. All other chemicals were from Sigma-Aldrich.

All constructs were cloned from the *MLSA1* or *MLSB* genes using either transfer polymerase chain reactions [Erijman et al., 2011; Erijman et al., 2014] or Gibson assembly [Gibson et al., 2009] and the primers described in Table S1. The products were treated with *Dpn*I for 2 h at 37 °C and transformed into competent *E. coli* DH5α cells (Life Technologies) prior to conducting colony polymerase chain reactions using the method of Nybo [Nybo, 2012] to filter out false positive colonies. Plasmid DNA was isolated from an overnight culture using a Wizard Mini-prep set (Promega). Final confirmation of cloning steps was obtained by DNA sequencing (DNA Sequencing Facility, Department of Biochemistry, University of Cambridge).

2. Protein expression and purification of KR domain samples

mKRa

The sequence coding for mKRa in module 5 of the *mlsA1* gene from *Mycobacterium ulcerans* (Uniprot: Q6MZA4; residues 10604-11078; Table S2) was cloned using the primers into pVB, a modified pET28α vector in which the amino acid recognition sequence for thrombin had been replaced with that for tobacco etch virus (TEV) protease, preceeded by an N-terminal His_6-tag fused to GB1, the 56-residue B1 immunoglobulin binding domain of Streptococcal protein G [Bao et al., 2006]. The pVB-mKRa plasmid was transformed into competent *E. coli* Tuner (DE3) cells (Merck). The His_6-GB1-mKRa fusion protein was expressed by growing the cells at 37 °C in 1 L of LB medium, prepared according to standard protocols [Sambrook & Russell, 2001], with 30 µg/mL kanamycin (Sigma) for selection, to a 600 nm optical density of 0.8, followed by induction with 0.5 mM isopropyl β-D-1-thiogalacto-pyranoside (IPTG; Sigma) and incubation at 20 °C for 20 h. Cells were harvested by centrifugation (20 min; 3583 x g), resuspended in lysis buffer (50 mM Na_2HPO_4, 300 mM NaCl, pH 8.0) with 5 mM imidazole, 2.5 units/mL benzonase nuclease (EMD Millipore) and Sigmafast EDTA-free protease inhibitor cocktail (Sigma) and then lysed using an Emulsiflex C5 homogeniser (Glen Creston). The clarified lysate was passed through Ni-NTA resin (Qiagen), washed twice with lysis buffer containing 30 mM imidazole and eluted with a buffer containing 50 mM Na_2HPO_4, 300 mM NaCl, 300 mM imidazole, 0.01 % (v/v) NaN_3, pH 8.0. The eluted protein was exchanged into phosphate buffer (50 mM Na_2HPO_4, 150 mM NaCl, 0.01 % (v/v) NaN_3, pH 7.5) and the His_6-GB1-tag was cleaved by overnight

incubation at 4 °C with 100 µL N-terminally His_6-tagged TEV protease in 1 mL TEV buffer (1 M Na_2HPO_4, 10 mM EDTA, 20 mM DTT, pH 7.5). The released His_6-GB1-tag was removed by passing the sample through Ni-NTA resin (Qiagen). The resin was flushed with a buffer containing 50 mM Na_2HPO_4, 300 mM NaCl, 5 mM imidazole, 0.01 % (v/v) NaN_3, pH 8.0 and then washed with 50 mM Na_2HPO_4, 300 mM NaCl, 30 mM imidazole, 0.01 % (v/v) NaN_3, pH 8.0; all the protein flow through was collected. The His_6-tagged GB1 and TEV protease were eluted using a buffer containing 50 mM Na_2HPO_4, 300 mM NaCl, 30 mM imidazole, 0.01 % (v/v) NaN_3, pH 8.0. The mKRa in the flow through was further purified by size exclusion chromatography using an Äkta Purifier 10 system equipped with a Superdex 75 10/300 column (GE Healthcare) in a buffer containing 25 mM HEPES, 50 mM NaCl. The mKRa sample was concentrated using 5000 MWCO Vivaspin 20 columns (Sartorius Stedim) and stored at 4 °C. All expression and purification steps were monitored by SDS-PAGE (NuPAGE) 4-12 % Bis-Tris gels (Life Technologies) stained with InstantBlue (Expedeon) (Figure S2).

mKRb

The sequence coding for mKRb in module 7 of the *mlsB* gene from *Mycobacterium ulcerans* (Uniprot: Q32YM8; residues 13784-13874; Table S2) was cloned into pVB and the resulting pVB-mKRb plasmid was transformed into competent *E. coli* Tuner (DE3) cells (Merck). The His_6-GB1-mKRb fusion protein was expressed and the released mKRb product was purified using the same protocol described above for mKRa. All expression and purification steps were monitored by SDS-PAGE (NuPAGE) 4-12 % Bis-Tris gels (Life Technologies) stained with InstantBlue (Expedeon) (Figure S1).

3. Protein expression and purification of *apo* ACP samples

mACPa

The sequence coding for mACPa in module 5 of the *MLSA1* gene from *Mycobacterium ulcerans* (Uniprot: Q6MZA4; residues 11087-11185; Table S2) was cloned into pVH, a modified pET28α vector in which the recognition sequence for thrombin had been replaced with that for tobacco etch virus (TEV) protease. The pVH-mACPa plasmid was transformed into competent *E. coli* Tuner (DE3) cells (Merck) and N-terminally His_6-tagged mACPa protein was expressed by growing the cells at 37 °C in 1 L of LB medium, prepared according to standard protocols [Sambrook & Russell, 2001], with 30 µg/mL kanamycin (Sigma) for selection, to a 600 nm optical density of 0.8, followed by induction with 0.5 mM isopropyl β-D-1-thiogalactopyranoside (IPTG; Sigma) and incubation at 15 °C for 16 h. Cells were harvested by centrifugation (20 min; 3583 x g), resuspended in lysis buffer (50 mM Na_2HPO_4, 300 mM NaCl, pH 8.0) with 5 mM imidazole, 2.5 units/mL benzonase nuclease (EMD Millipore) and Sigmafast EDTA-free protease inhibitor cocktail (Sigma) and lysed using an Emulsiflex

C5 homogeniser (Glen Creston). The clarified lysate was passed through Ni-NTA resin (Qiagen), washed twice with lysis buffer containing 30 mM imidazole and eluted with lysis buffer containing (50 mM Na_2HPO_4, 300 mM NaCl, 300 mM imidazole, 0.01 % (v/v) NaN_3, pH 8.0). The eluted protein was exchanged into phosphate buffer (50 mM Na_2HPO_4, 150 mM NaCl, pH 7.5) and the His_6-tag was cleaved by overnight incubation at 4 °C with TEV protease in 1 mL TEV buffer (1 M sodium phosphate, 10 mM EDTA, 20 mM DTT, pH 7.5). The released mACPa was further purified by size exclusion chromatography using an Äkta Purifier 10 system equipped with a Superdex 75 10/300 column (GE Healthcare) in phosphate buffer. The sample was concentrated using 5000 MWCO Vivaspin 20 columns (Sartorius Stedim). All expression and purification steps were monitored by SDS-PAGE (NuPAGE) 4-12 % Bis-Tris gels (Life Technologies) stained with InstantBlue (Expedeon) (Figure S2). The identity of the sample was confirmed by electrospray injection mass spectrometry (ESI-MS; PNAC facility, Department of Biochemistry, University of Cambridge; Table S3 and Figure S3).

mACPb

The amino acid sequence for mACPb coded in module 7 of the *MLSB* gene from *Mycobacterium ulcerans* (Uniprot: Q32YM8; residues 13784-13874; Table S2) is identical to that of the ACP domain ($mACP_9$) coded by the *MLSA2* gene (Uniprot: Q6MZA5; residues 2050-2140) studied previously by Vance and coworkers [Vance et al., 2016]. As described in that paper, a pET28α vector was used to express N-terminally His_6-tagged mACPb in *E. coli* Tuner (DE3) cells (Merck). The cells were grown at 37 °C in 1 L of LB medium, prepared according to standard protocols [Sambrook & Russell, 2001], with 30 µg/mL kanamycin (Sigma) for selection, to a 600 nm optical density of 0.8, followed by induction with 0.5 mM isopropyl β-D-1-thiogalactopyranoside (IPTG; Sigma) and incubation at 15 °C for 16 h. The cells were harvested and mACPb was purified and concentrated as described above for mACPa, except His_6-tag cleavage was performed using restriction grade thrombin (EMD Millipore). All expression and purification steps were monitored by SDS-PAGE (NuPAGE) 4-12 % Bis-Tris gels (Life Technologies) stained with InstantBlue (Expedeon) (Figure S2) and the identity of the sample was confirmed by ESI-MS (PNAC facility, Department of Biochemistry, University of Cambridge; Table S3 and Figure S3).

4. Validation of KR domain secondary structure and reductase activity

Despite extensive trials, purified samples of mKRa and mKRb proved to be unsuitable for analysis by mass spectrometry using the Waters Q-TOF micro system in the Protein and Nucleic Acid (PNAC) Facility, Department of Biochemistry, University of Cambridge, due to aggregation during the injection step. To validate these samples, we confirmed that the enzyme domains possessed secondary

structure using circular dichroism (CD) spectroscopy and displayed NADPH-dependent reductase activity against *trans*-1-decalone using a previously established assay [Siskos et al., 2005].

For CD spectroscopy, protein samples were prepared at 0.2 mg/mL in phosphate buffer (45 mM Na_2HPO_4, 5 mM NaH_2PO_4, 150 mM NaF, 0.01 % (v/v) NaN_3, pH 7.5). Molar ellipticity data, [θ], was collected at 30 °C using an Aviv model 410 circular dichroism spectrometer between 200 and 260 nm. Runs acquired in triplicate were used to calculate the average spectra displayed in Figure S5, which indiacte that both mKRa and mKRb contain a significant proportion of α-helical secondary structure. For comparison, the X-ray structure of the KR domain from module 1 of the erythromycin polyketide synthase (PDB code, 2FR0) possesses an α-helix content of 37% [Keatinge-Clay & Stroud, 2006].

KR domain activities were assessed using a previously designed assay by incubation at 30 °C with NADPH and the substrate mimic *trans*-1-decalone (TDL) in HEPES buffer (25 mM HEPES, 50 mM NaCl, pH 7.5) at 30 °C. Assay reactions were set up in a Costar 96 well UV-transparent plate (Corning) and the progress of the reaction was followed for 20 min by monitoring the depletion of NADPH at 340 nm at 20 s intervals using a Spectramax Plus microplate reader running Softmax Pro (Molecular Devices) with 2 s shaking in between reads. The final assay volume was 110 µL, yielding NADPH and KR domain concentrations of 1.3 mM and 8 µM, respectively. The KR domain was incubated with NADPH for 20 min prior to addition of TDL. TDL was dissolved in 3 % (v/v) DMSO and its initial well concentration was varied from 0 to 50 mM, while the total amount of DMSO in the well was kept constant. Assays were performed in triplicate for each run to generate plots of the initial rate of consumption of NADPH (v_0) against TDL concentration. Fitting to the Michaelis-Menten equation, $v_0 = V_{max}[TDL]/(K_M + [TDL])$, using SoftmaxPro v6 yielded values for V_{max}, k_{cat} and K_M, as displayed in Table S4. These results confirm that the KR domain constructs prepared for this work possess kinetic parameters very similar to those obtained in previous studies [Bali & Weissman, 2006].

Overall, since both mKRa and mKRb contain appropriate secondary structure and show activities similar to those of previously studied equivalents, we concluded that our samples are likely to possess native tertiary structure.

5. Preparation of *holo* and acyl-loaded ACP samples

ACP constructs were prepared in the Ppant-attached *holo* form *in vivo* by co-expression. Two expression vectors, one coding for the ACP construct (pVH-mACPa or pET28α-mACPb) and the other coding for the broad specificity phosphopantetheinyl transferase Sfp (pET-Sfp) [Quadri et al., 1998], were co-transformed into *E. coli* Tuner(DE3) cells. Procedures for the expression and purification of the *holo* ACP species were as described for the *apo* species in Section 3 above. The modification state

of the ACP domain was confirmed by ESI-MS (PNAC facility, Department of Biochemistry, University of Cambridge; Table S3 and Figure S3).

Loading reactions for acyl-ACP species were set up *in vitro*. An *apo* ACP sample (0.1 mM) was incubated at 27 °C for 2 h with Sfp (4.4 mM) and either acetoacetyl-CoA (2 mM) or β-hydroxybutyryl-CoA (2 mM) in phosphate buffer (45 mM Na_2HPO_4, 5 mM NaH_2PO_4, 150 mM NaCl, 0.01 % (v/v) NaN_3, pH 7.5) supplemented with 10 mM $MgCl_2$. To separate the loaded protein from Sfp and any excess substrate, the mixture was purified by size exclusion chromatography, as described in Section 3 above. The identity of the eluted protein was confirmed by ESI-MS (PNAC facility, Department of Biochemistry, University of Cambridge; Table S3 and Figure S4). For acyl-ACP samples, ESI-MS analysis was carried out after conducting the ITC experiments described in Section 6 below. Although the samples were stored at 4 °C during this 24 h period, both of the acetoacetyl-ACP species proved to be mildly unstable: their mass spectra in panels A and B of Figure S3 show minor species that result from hydrolysis to the *holo* ACP form. However, the absence of signals from the starting material (*apo* ACP species) indicate that in both cases the loading reaction had run to completion. By contrast, no hydrolysis was observed for the two β-hydroxybutyryl-ACP species (panels C and D of Figure S3).

6. Isothermal titration calorimetry experiments

ITC measurements of affinity (K_D), stoichiometry (n) and apparent enthalpy change ($\Delta H°$) were obtained using a VP-ITC microcalorimeter (MicroCal Inc.). Samples for the cell and injectant solutions were prepared in the same buffer (25 mM HEPES, 50 mM NaCl, 0.01 % (v/v) NaN_3, pH 7.5). Small compounds of interest were dissolved in the flow through from the 5 kDa MWCO Sartorius centrifugal concentrators used to prepare each KR domain sample, to ensure that the buffers were closely matched. All samples were degassed under vacuum using a ThermoVac accessory (MicroCal Inc.) and loaded into the cell and the syringe. The same protocol was used for all experiments, which were conducted at 30 °C with a stirring speed of 300 rpm and a sequence of 29 injections of 10 µL, each lasting 7.1 s, and with a 240 s interval between each injection. The first injection was set to a smaller volume (2 µL in 3.3 s) to allow the cell and needle solutions to mix, and the resulting heat change was disregarded in the later analysis. Control experiments were run to confirm that dilution heat changes caused by the titration of injectant into the cell solution were negligible. Dilution heat change thermograms for each cell sample were subtracted from the final trace before integration with respect to time to generate an isotherm trace. All isotherm traces were analysed using ORIGIN, version 7.0 (MicroCal, Inc.) with a model that assumed a single binding site. The resulting K_D values were averaged across 3 technical replicates and a standard deviation was calculated. Representative ITC

thermograms and isotherm plots for experiments with KR samples in the cell and either ACP species or Ppant fragments in the syringe are displayed in Figures S6, S7, S8 and S9.

Interestingly, the heat changes observed on titration of either *apo* mACPa or *apo* mACPb against their cognate KR domains in the absence of NADPH or $NADP^+$ were miniscule (data not shown); at ± 0.1 µcal s^{-1} per injection, these responses were little different from those observed in dilution control experiments (see Fig. 2 of the main text). The lack of a strong heat change signature in the absence of cofactor indicates that binding is weak or non-existent, or that the interaction between the injectant and its binding partner must be isothermic under the conditions of the experiment. Furthermore, in the absence of cofactor titration of *holo* or acyl-ACP species, or of any of the prosthetic group fragments detailed in Scheme 2 of the main text, against either of the KR domains again produced thermograms that resembled dilution control experiments (data not shown). Finally, titration of NADPH or $NADP^+$ against either of the KR domains yielded similarly weak heat changes (data not shown). Since there is no doubt that the cofactor must bind to the enzyme so that ketoreduction can occur, we conclude that these interactions must be isothermic under the experimental conditions employed here.

Table S1: Oligonucleotide primers

PCR primers used for cloning the mycolactone polyketide synthase KR and ACP constructs.

Target	Direction	DNA sequence (5' to 3')
mKRa	F	AGCAGCGGCGAAAACCTGTATTTTCAGGGCCGCGCGGAGCGCA
	R	GCCAACTCAGCTTCCTTTCGGGCTTTGTTAGCGGTAAGCCATTACCTCTGTG
mKRb	F	AGCAGCGGCGAAAACCTGTATTTTCAGGGCGACTCATTGATCACCCGCCCC
	R	ACCACACCACGGCGCCGGTAACAAAGCCCGAAAGGAAGCTGAGTTGGC
mACPa	F	AGCAGCGGCGAAAACCTGTATTTTCAGGGCTCGACGGCGACGCTCC
	R	GCCAACTCAGCTTCCTTTCGGGCTTTGTTAACCGATGCCAGGGATCTGTTC
mACPb	F	AGCAGCGGCGAAAACCTGTATTTTCAGGCGCCTCAACGGACTTAGC
	R	GCCAACTCAGCTTCCTTTCGGGCTTTGTTAATGGCTTTGGGTGAGTCG

Table S2: Amino acid sequences of protein constructs

Amino acid sequences of the mycolactone polyketide synthase KR and ACP constructs.

Construct	Amino acid sequence (N-terminal to C-terminal)
mKRa	GRAERTQSEL DSWRYQVTWL SSPATPSSIT LSGIWLLIVP SELAKTDPVI GCAAALEAHG ALVTIITIFE PDFNRSLMGA SLKDIGSHIS GVISFLGIHG SEFSDSGAVK TLNLVQAMGD VHLDVPLWCL TQGAVSISAD DLIRCSSAAL VWGLGRVVAL EHPGSWGGLV DLPESPDDAA WERLCALLAQ PTDEDQFAIR PSGVFLRRLI HAPATTTSKS STAWAPRGTV LITGGTGALG AHVARWLAHK YESVDLLLTS RRGMAADGAT ELVDDLRTAG ASVTVHACDV TDRTSVEAAI AGKSLDAVFH LAGRHQPTLL TELEDESFSD ELAPKVHGAQ VLSDITSNLT LSAFVMFSSV AGIWGGKSQG AYAAANAFLD SLAEKRRTLG LPATSVAWGL WAGGGMGDRP SASGLNLIGL KSMSADLAVQ ALSDAIDRPQ ATLTVASVNW DRFYPTFALA RPRPFLHEIT EVMAYR
mKRb	GDSLITRPLT TATGSAPATT AAGLLHLSWP PHPDTTTDTD TDTDALRYQV IAEPTQQLPR YLHDLHTSTD LHTSTTEADV VVWPVPVPSN EELQAHQASD TAVSSRIHTL TRQTLTVVQD WLTHPDTTGT RLVIVTRHGV STSAHDPVPD LAHAAVWGLI RSAQNEHPGR FTLLDTDDNT NSDTLTTALT LPTRENQLAI RRDTIHIPRL TRHSSDGALT APVVVDPEGT VLITGGTGTL GALFAEHLVS AHGVRHLLLT SRRGPQAHGA TDLQQRLTDL GAHVTITACD ISDPEALAAL VNSVPTQHRL TAVVHTAAVL ADTPVTELTG DQLDQVLAPK IDAAWQLHQL TYEHNLSAFI MFSSMAGMIG SPGQGNYAAA NTALDALADY RHRLGLPATS LAWGYWQTHT GLTAHLTDVD LARMTRLGLM PIATSHGLAL FDAALATGQP VSIPAPINTH TLARHARDNT LAPILSALIT TPRRR
mACPa	GSTATLLTSK LAGLTATEQR AVTRKLVLDQ AASVLGYAST ESLDTHESFK DLGFDSLTAL ELRDHLQTAT GLNLSSTLIF DHPTPHAVAE HLLEQIPGIG
mACPb	GSHMRLNGLS PQQQQTLAT LVAAATATVL GHHTPESISP ATAFKDLGID SLTALELRNT LTHNTGLDLP PTLIFDHPTP HALTQHLHTR LTQSH

Table S3: ESI-MS analysis of ACP species

Predicted and experimental molecular masses for ACP species determined by ESI-MS.

**ACP species**	**Predicted mass / Da**	**Experimental mass / Da**
apo mACPa	10596.1	10598.0
holo mACPa	10937.2	10937.3
acetoacetyl-mACPa	11021.2	11021.3
β-hydroxybutyryl-mACPa	11023.2	11023.2
apo mACPb	10224.5	10224.8
holo mACPb	10564.8	10564.8
acetoacetyl-mACPb	10648.8	10649.6
β-hydroxybutyryl-mACPb	10650.8	10650.8

Table S4: Kinetic parameters for KR domain constructs

Comparison of kinetic parameters determined for mycolactone KR domain species in the current work with those obtained previously [Bali & Weissman, 2006]. Data shows values for the mean and the standard deviation obtained from three technical replicate experiments.

**KR species**	K_M / mM	k_{cat} / s^{-1}	k_{cat}/K_M / $s^{-1} M^{-1}$
mKRa (current work)	3.0 (± 0.4)	0.20 (± 0.002)	66 (± 22)
mKRa (previous)	3.2 (± 0.5)	0.28 (± 0.01)	88 (± 17)
mKRb (current work)	3.1 (± 0.7)	0.40 (± 0.01)	129 (± 14)
mKRb (previous)	3.7 (± 0.4)	0.52 (± 0.02)	140 (± 20)

Figure S1: Module organization for the mycolactone PKS system

Module organization for the three subunits of the mycolactone PKS system (MLSA1, MLSA2 and MLSB) [Stinear et al., 2004]. The structure of mycolactone is colour coded to match the subunits responsible for synthesizing each segment. A1-and B1-type KR domains are represented in white and magenta, respectively. DH domains that are predicted to be inactive are marked with diagonal black lines. Domain abbreviations: KS, ketosynthase; AT, acyltransferase; KR, ketoreductase; DH, dehydratase; ER, enoyl reductase; CP, acyl carrier protein; TE, thioesterase.

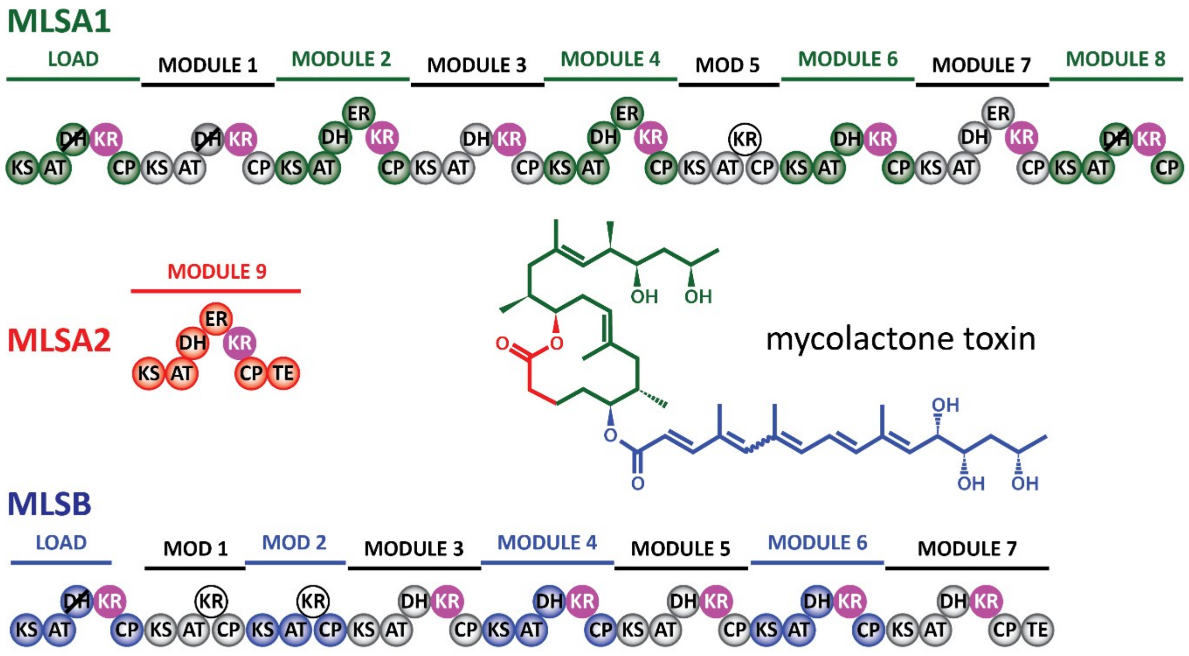

.

Figure S2: SDS-PAGE analysis

Each panel displays a 4-12 % Bis-Tris gel containing Thermo Scientific PageRuler prestrained protein ladder (left lane) and the indicated protein construct after purification by size exclusion chromatography (right lane).

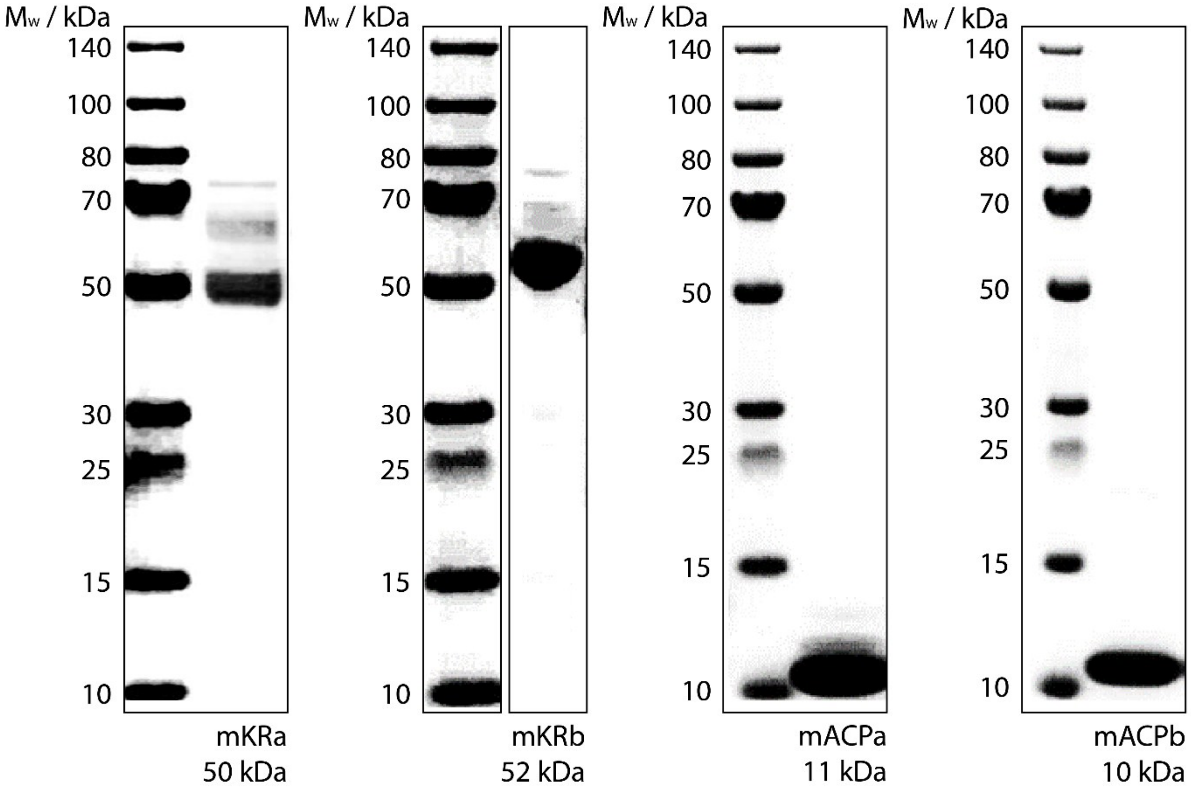

Figure S3: ESI-MS spectra of *apo* and *holo* ACP species

Deconvoluted electrospray mass spectra for samples of: (A) *apo* mACPa; (B) *apo* mACPb; (C) *holo* mACPa; and (D) *holo* mACPb.

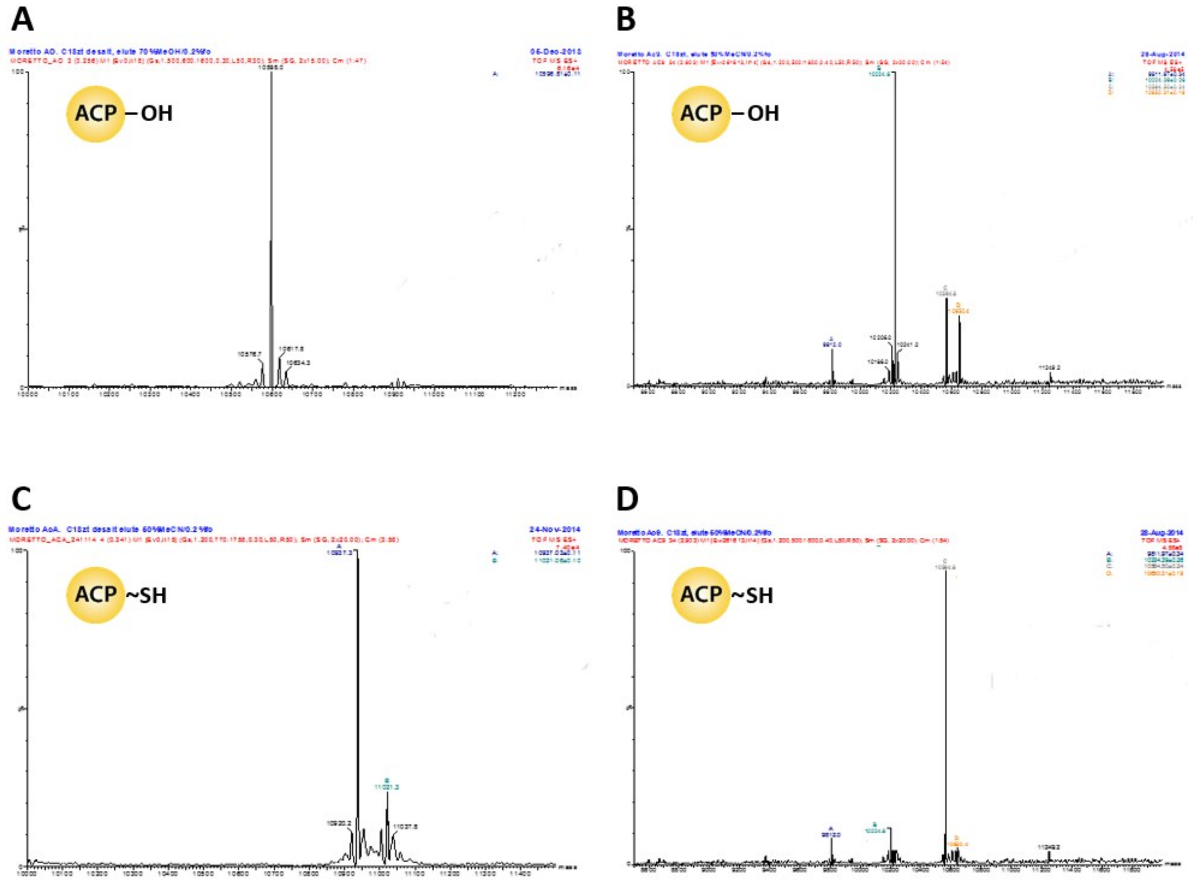

Figure S4: ESI-MS spectra of acyl-ACP species

Deconvoluted electrospray mass spectra for samples of: (A) acetoacetyl-mACPa; (B) acetoacetyl-mACPb; (C) β-hydroxybutyryl-mACPa; and (D) β-hydroxy-butyryl-mACPb.

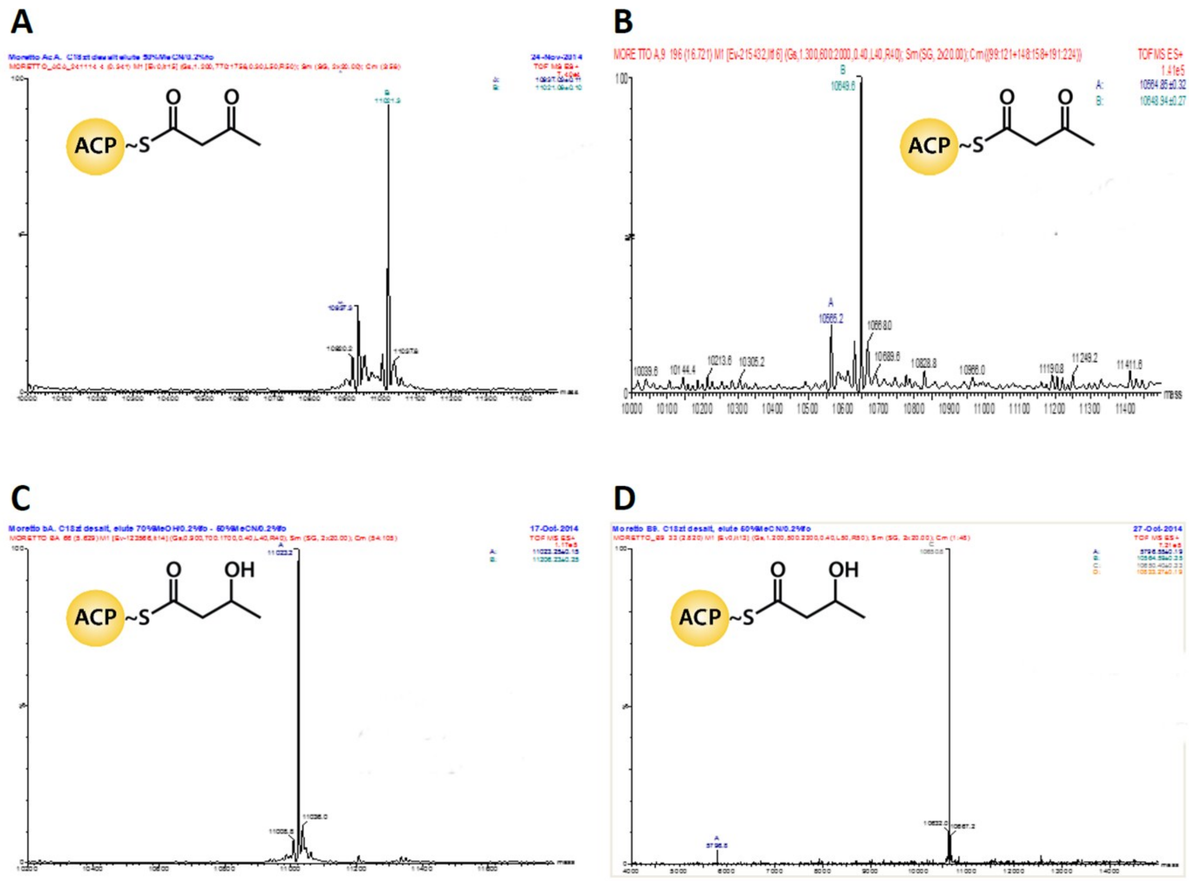

Figure S5: Circular dichroism spectra of KR domain constructs

CD spectra are displayed for samples of (A) mKRa and (B) mKRb, both showing minima at 209 nm and 222 nm, which are characteristic α-helical secondary structure.

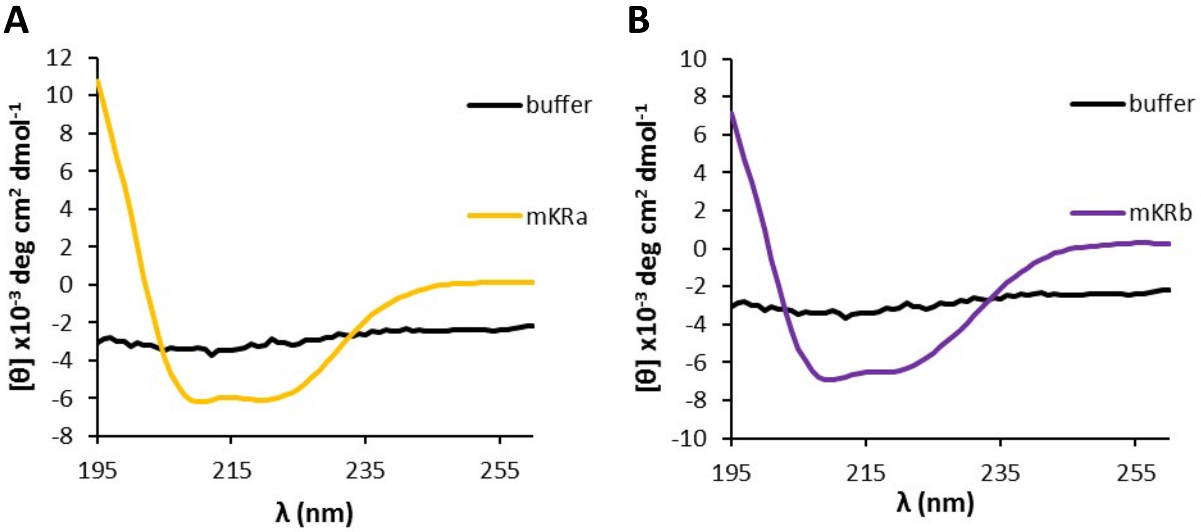

Figure S6: Isothermal titration calorimetry data I

Representative ITC thermograms (upper panels) and isotherm plots (lower panels), all in the presence of excess NADPH, showing consecutive injections of: (A) *apo* mACPa against mKRb; (B) *apo* mACPb against mKRa; (C) *holo* mACPa against mKRa; (D) *holo* mACPb against mKRb; (E) β-hydroxybutyryl-mACPa against mKRa; and (F) β-hydroxybutyryl-mACPb against mKRb. Thermogram traces for dilution control experiments are shown at the top of each upper panel.

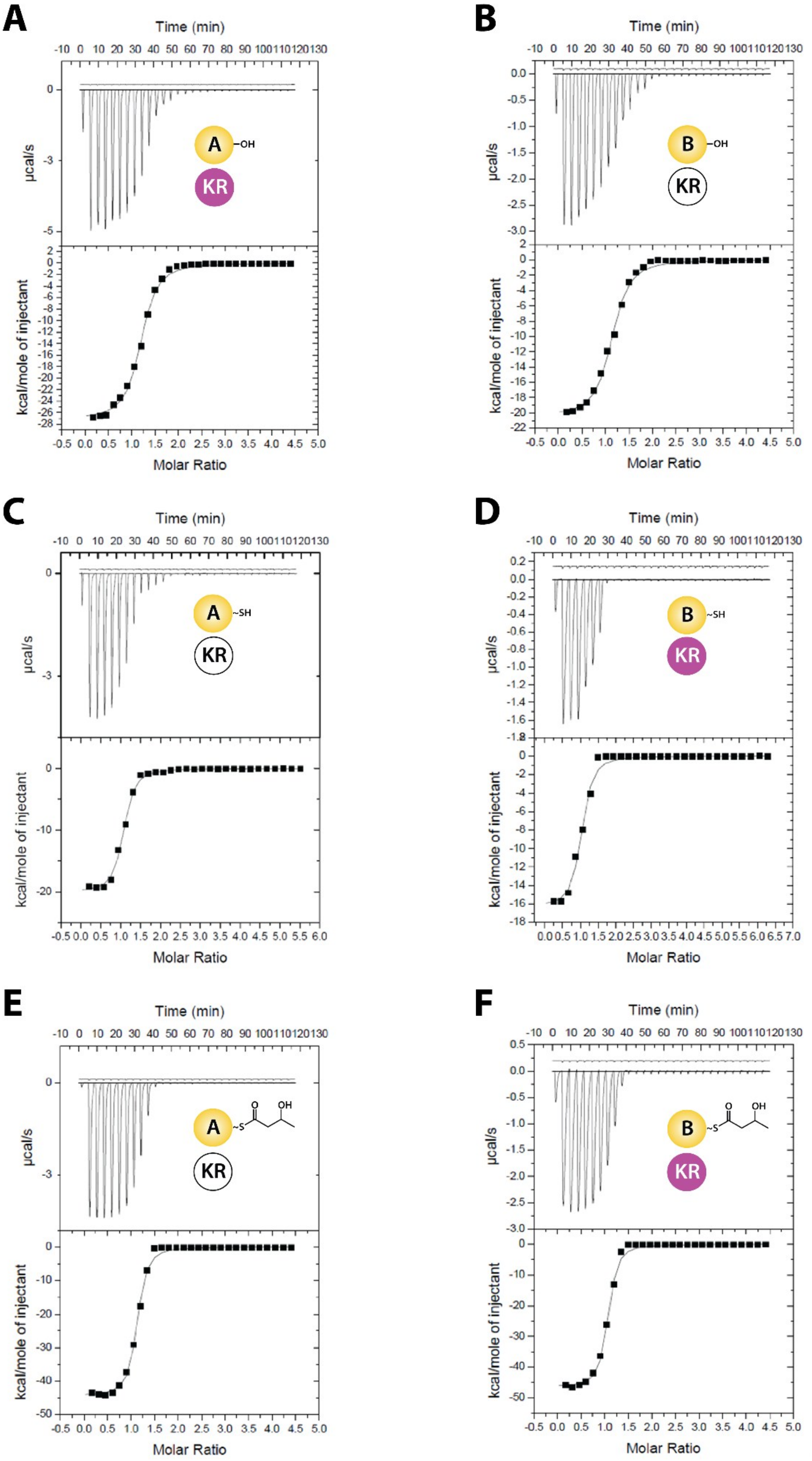

Figure S7: Isothermal titration calorimetry data II

Representative ITC thermograms (upper panels) and isotherm plots (lower panels), all in the presence of excess $NADP^+$, showing consecutive injections of: (A) *apo* mACPa against mKRa; (B) *apo* mACPb against mKRb; (C) *holo* mACPa against mKRa; (D) *holo* mACPb against mKRb; (E) acetoacetyl-mACPa against mKRa; and (F) acetoacetyl-mACPb against mKRb. Thermogram traces for dilution control experiments are shown at the top of each upper panel.

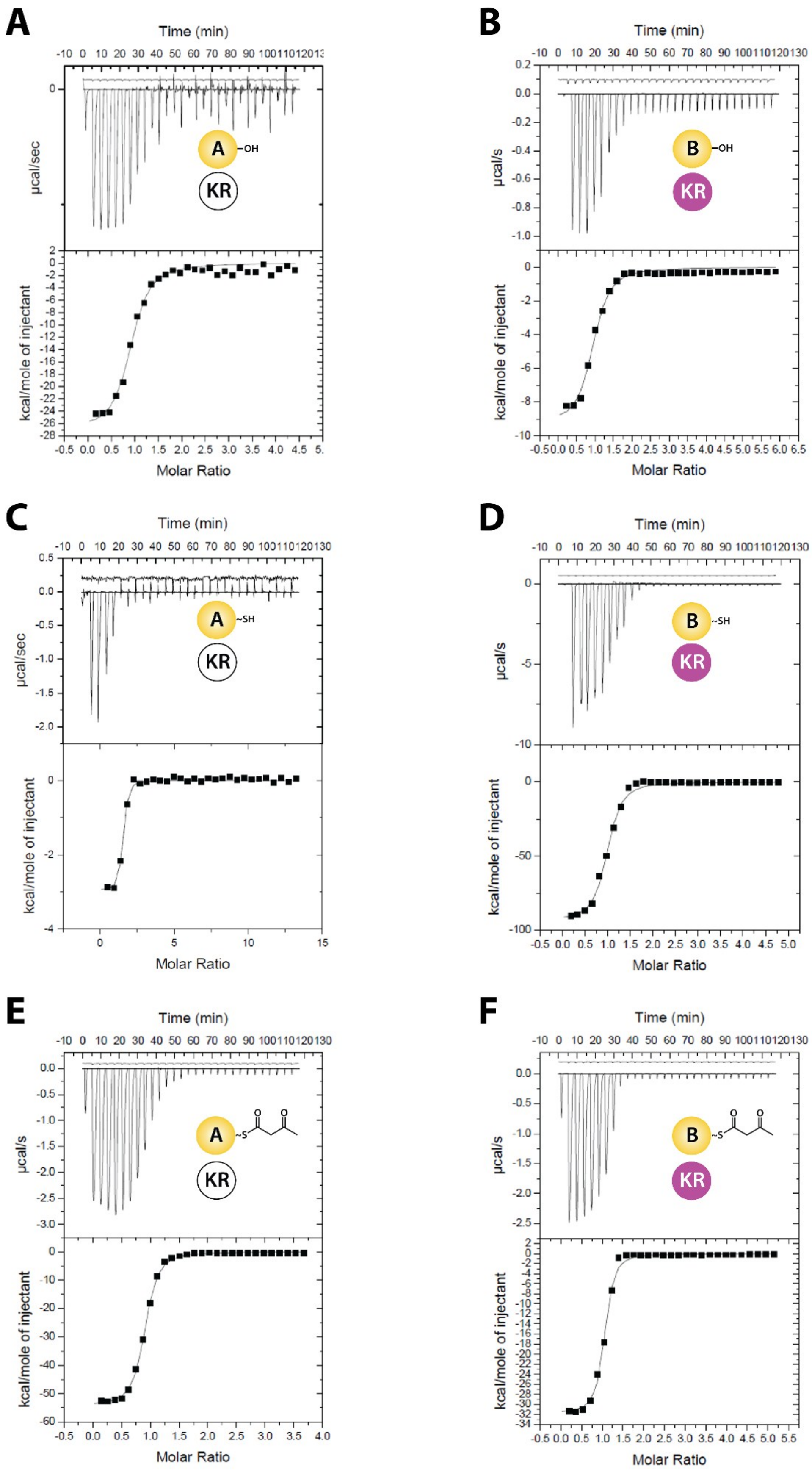

Figure S8: Isothermal titration calorimetry data III

Representative ITC thermograms (upper panels) and isotherm plots (lower panels), all in the presence of excess NADPH, showing consecutive injections of: (A) R-pantetheine against mKRa; (B) R-pantetheine against mKRb; (C) pantoate against mKRa; (D) pantoate against mKRb; (E) β-alanine against mKRa; (F) β-alanine against mKRb; (G) *N*-acetyl cysteamine against mKRa; (H) *N*-acetyl cysteamine against mKRb; and (I) cysteamine against mKRb. Thermogram traces for dilution control experiments are shown at the top of each upper panel.

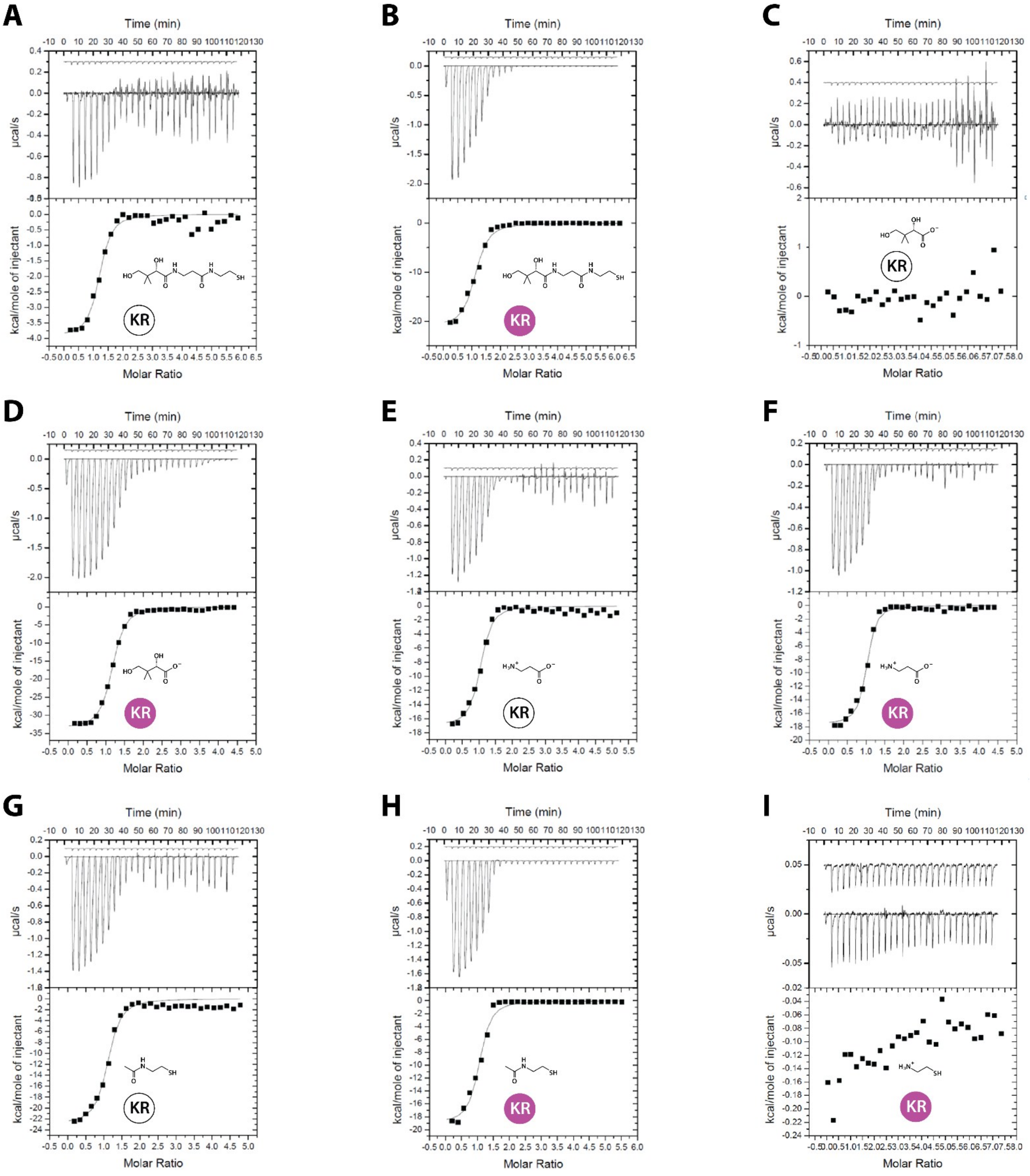

Figure S9: Isothermal titration calorimetry data IV

Representative ITC thermograms (upper panels) and isotherm plots (lower panels), all in the presence of excess NADP$^+$, showing consecutive injections of: (A) R-pantetheine against mKRa; (B) R-pantetheine against mKRb; (C) pantoate against mKRa; (D) pantoate against mKRb; (E) β-alanine against mKRa; (F) β-alanine against mKRb; (G) *N*-acetyl cysteamine against mKRa; (H) *N*-acetyl cysteamine against mKRb; and (I) cysteamine against mKRb. Thermogram traces for dilution control experiments are shown at the top of each upper panel.

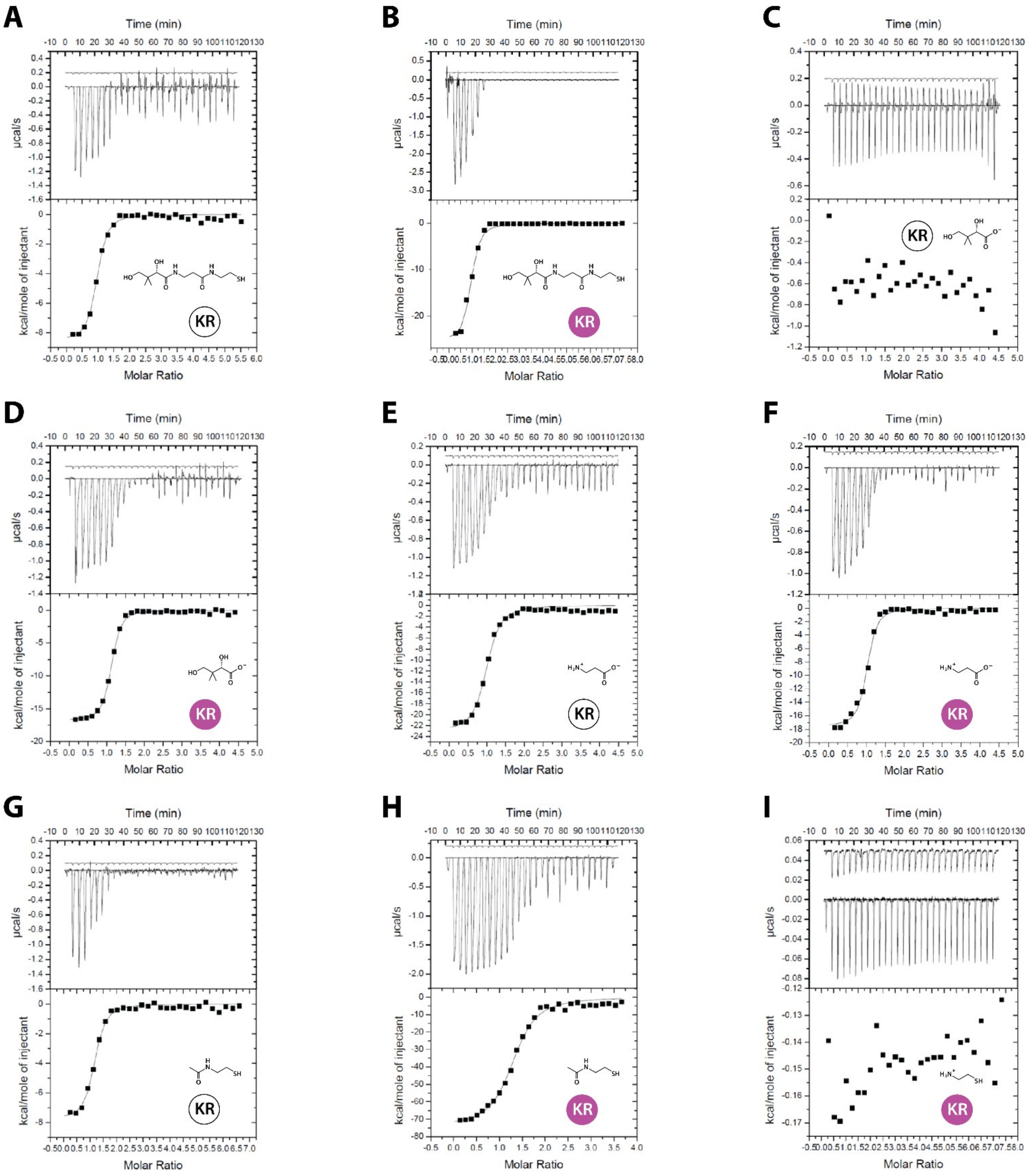

7. References

Bali, S., Weissman, K.J. (2006). Ketoreduction in mycolactone biosynthesis: insight into substrate specificity and stereocontrol from studies of discrete ketoreductase domains *in vitro*. *ChemBioChem*, **7**, 1935–42.

Bao, W., Gao, Y., Chang. Y., Zhang, T., Lin, X., Yan, X., Hu, H. (2006) Highly efficient expression and purification system of small-size protein domains in *Escherichia coli* for biochemical characterization. *Protein Expr. Purif.*, **47**, 599–606.

Erijman, A., Dantes, A., Bernheim, R., Shifman, J. M., Peleg, Y. (2011) Transfer-PCR (TPCR): a highway for DNA cloning and protein engineering. *J. Struct. Biol.*, **175**, 171–7.

Erijman, A., Shifman, J. M., Peleg, Y. (2014). DNA Cloning and Assembly Methods. *Springer*, *1116*, 89–101.

Gibson, D.G., Young, L., Chuang, R.-Y., Venter, J.C., Hutchison, C.A., Smith, H.O. (2009). Enzymatic assembly of DNA molecules up to several hundred kilobases. *Nature Methods*, **6**, 343–345.

Keatinge-Clay, A.T., Stroud, R.M. (2006) The structure of a ketoreductase determines the organization of the beta-carbon processing enzymes of modular polyketide synthases. *Structure*, **14**, 737-748.

Nybo, K. (2012). Colony PCR. *BioTechniques*, **53**, 72.

Quadri, L.E., Weinreb, P.H., Lei, M., Nakano, M.M., Zuber, P., Walsh, C.T. (1998) Characterization of Sfp, a *Bacillus subtilis* phosphopantetheinyl transferase for peptidyl carrier protein domains in peptide synthetases. *Biochemistry*, **37**, 1585–1595.

Sambrook, J., Russell, D.W. (2001) Molecular Cloning: A Laboratory Manual. Cold Spring Harbor Laboratory Press. 3rd edn.

Siskos, A.P., Baerga-Ortiz, A., Bali, S., Stein, V., Mamdani, H., Spiteller, D., Popovic, B., Spencer, J.B., Staunton, J.,Weissman, K.J., Leadlay, P.F. (2005). Molecular basis of Celmer's rules: stereochemistry of catalysis by isolated ketoreductase domains from modular polyketide synthases. *Chem. Biol.*, **12**, 1145–1153.

Stinear, T.P., Mve-Obiang, A., Small, P.L., Frigui, W., Pryor, M.J., Brosch, R., Jenkin, G.A., Johnson, P.D., Davies, J.K., Lee, R.E., Adusumilli, S., Garnier, T., Haydock, S.F., Leadlay, P.F., Cole, S.T. (2004) Giant plasmid-encoded polyketide synthases produce the macrolide toxin of *Mycobacterium ulcerans*. *Proc. Natl. Acad. Sci. USA*, **101**, 1345-1249.

Vance, S., Tkachenko, O., Thomas, B., Bassuni, M., Hong, H., Nietlispach, D. & Broadhurst, R.W. (2016) The dynamics of a sticky swinging arm: studies of an acyl carrier protein domain from the mycolactone polyketide synthase. *Biochem. J.*, **473**, 1097-1110.
